# Diagnosing Depression in Chronic Pain Patients: DSM-IV Major Depressive Disorder vs. Beck Depression Inventory (BDI)

**DOI:** 10.1371/journal.pone.0151982

**Published:** 2016-03-23

**Authors:** Peter Knaster, Ann-Mari Estlander, Hasse Karlsson, Jaakko Kaprio, Eija Kalso

**Affiliations:** 1 Pain Clinic, Department of Anaesthesiology, Intensive Care and Pain Medicine, Helsinki University Hospital and University of Helsinki, Helsinki, Finland; 2 Department of Clinical Science, University of Turku, Turku, Finland; 3 Faculty of Medicine and Institute for Molecular Medicine, University of Helsinki, Helsinki, Finland; Medical University of Vienna, AUSTRIA

## Abstract

**Background:**

Diagnosing depression in chronic pain is challenging due to overlapping somatic symptoms. In questionnaires, such as the Beck Depression Inventory (BDI), responses may be influenced more by pain than by the severity of depression. In addition, previous studies have suggested that symptoms of negative self-image, a key element in depression, are uncommon in chronic pain-related depression. The object of this study is to assess the relationship of the somatic and cognitive-emotional items of BDI with the diagnosis of depression, pain intensity, and disability.

**Methods:**

One hundred consecutive chronic pain patients completed the Structured Clinical Interview for DSM Disorders (SCID) for the diagnosis of major depressive disorder (MDD) according to DSM-IV. Two subscales of BDI (negative view of self and somatic-physical function) were created according to the factor model presented by Morley.

**Results:**

In the regression analysis, the somatic-physical function factor associated with MDD, while the negative view of self factor did not. Patients with MDD had higher scores in several of the BDI items when analysed separately. Insomnia and weight loss were not dependent on the depression diagnosis.

**Limitations:**

The relatively small sample size and the selected patient sample limit the generalisability of the results.

**Conclusions:**

Somatic symptoms of depression are also common in chronic pain and should not be excluded when diagnosing depression in pain patients. Regardless of the assessment method, diagnosing depression in chronic pain remains a challenge and requires careful interpretation of symptoms.

## Introduction

Depression is commonly observed to coexist with chronic pain. It is markedly associated with higher levels of reported pain, as well as with increased functional impairment [1–3]. Assessing depression in chronic pain is made challenging by symptom overlap. Symptoms such as insomnia, fatigue, and change in activity can be related to both pain and depression. According to DSM-IV, symptom criteria that are fully attributable to the medical condition should not be included in the psychiatric diagnosis [4]. However, there is no consensus on the treatment of these items in the context of various medical illnesses. Determining the etiology of a specific symptom is difficult or impossible [5–9]. Various diagnostic strategies, such as etiological, inclusive, exclusive or substitutive, may thus suggest differing rates of depression in medically ill patients [6,10].

The widely used Beck Depression Inventory (BDI) [11] was originally designed to measure the level of depression in patients already having that diagnosis. The validity of assessing symptoms of depression in medical illnesses using self-report questionnaires such as BDI has been questioned. [5,12–15]. Several items in the BDI can be attributed to the medical illness, e.g. sleep problems, difficulties with concentration, and fatigue, which may spuriously increase the sum score of the questionnaire. Therefore, the conventional cut-off scores for mild (10–18), moderate (19–29), and severe (30–63) depression, may not be valid for patients with somatic conditions [11,14–16].

Morley and colleagues [17] analysed 1947 chronic pain patients using the original BDI [11], presenting a resolution of its 21 items into two specific factors, the negative view of the self and the somatic and physical function-factor. A number of emotional items, such as sadness, pessimism or suicidal ideas, did not load on either factor. They suggested that the symptom pattern of depression in chronic pain patients differs from the psychiatric model of depression. Additionally, the BDI is likely to measure more general distress-related symptoms than depression in chronic pain [17]. Some of the core cognitive symptoms of depression such as guilt, worthlessness, and self-dislike may be less frequent in chronic pain-related depression than in psychiatric depression [18].

In the present study, the aim was to analyse the association of chronic pain and depression. The specific goal was to assess how the somatic and cognitive-emotional aspects of depression measured by the Beck Depression Inventory (BDI) are associated with the diagnosis of depression, based on the Structured Clinical Interview for DSM-IV Diagnosis (SCID) [19]. In order to determine a difference between the somatic and cognitive-emotional aspects of depression in chronic pain, we utilised the BDI model of Morley and colleagues [17].

## Methods

### Patients

One hundred chronic pain patients participated in the study. A total of 121 consecutive patients referred for assessment and treatment to the Helsinki University Central Hospital Pain Clinic were invited to participate during a scheduled visit to the clinic. Inclusion criteria were: aged from 30 to 60 years; chronic pain for at least one year; and fluency in the Finnish language. The exclusion criteria were: malignancy; medication with strong opioids; psychosis: and current drug or alcohol abuse. Eighteen patients chose not to participate. Three patients were excluded because of missing data. The study was approved by the Ethics Committee of the Helsinki University Hospital. All patients provided written informed consent.

### Assessment of Depressive Symptoms

The Beck Depression Inventory [11,20] is a 21-item self-administered scale measuring various symptoms of depression. It comprises 21 groups of statements describing the somatic and cognitive-emotional symptoms of depression. Each item consists of four alternative responses graded from 0 to 3 according to the severity of the symptom. The patients choose the response closest to their state during the past week. A sum score is counted, a higher score indicating more severe depression. A number of studies support the validity and other psychometric properties of the BDI in psychiatric patients [21–25]. In the current analysis, we utilised the recommendations by Morley and colleagues [17] and formed two subscales of the BDI: negative view of self, and somatic/physical function, reflecting the cognitive-emotional and somatic aspects of depression. The negative view of self scale (range 0–18) comprises six items related to sense of failure, guilt, punishment, self-dislike, self-accusation, and body image changes. The somatic/physical function scale (7 items, range 0–21) includes ratings of social withdrawal, work difficulty, insomnia, fatigability, loss of appetite, somatic preoccupation, and loss of libido. The remaining eight BDI items did not form any coherent factor in Morley and colleagues’ factor solution. These will be referred to as “items not included in the model” throughout the analyses.

### SCID DSM-IV

The psychiatric assessment was performed using the Structured Clinical Interview for DSM-IV Axis 1 disorders (SCID-I) [19], with a trained interviewer (PK). The diagnosis of Major Depressive Disorder (MDD) requires that five or more of the following symptoms have been present during the same 2-week period and represent a change from previous functioning. At least one of the symptoms is either (1) depressed mood or (2) loss of interest or pleasure: these are considered the core symptoms of the disorder. The other criteria are (3) significant weight loss or gain, (4) insomnia or hypersomnia, (5) psychomotor agitation or retardation, (6) fatigue or loss of energy nearly every day, (7) feelings of worthlessness or excessive or inappropriate guilt, (8) diminished ability to think or concentrate, or indecisiveness, and (9) recurrent thoughts of death.

### Pain Measurements

The Pain Questionnaire (in Finnish “Kipukysely”, www.suomenkivuntutkimusyhdistys.fi/), a routine self-administered questionnaire for all patients at the Helsinki University Central Hospital Pain Clinic, was used. Demographic information and the current pain intensity measurement using the Visual Analogue Scale (VAS) were extracted from this questionnaire. Pain intensity was assessed using the Visual Analogue Scale (VAS), a 10 cm horizontal line with 0 representing no pain and 10 maximum possible pain. The patients were asked to mark on the line an estimate of their current pain intensity. The questionnaire includes a pain disability section, comprising18 items. Each item has three options; 1 “not at all”, 2 “somewhat”, and 3 “much”. The question “does the pain interfere with the following activities?” is followed by a list of daily activities such as lying, sitting, standing, cleaning, reading, sleeping, driving, social activities, sexual activities etc. A sum score is calculated, a higher score indicating more severe disability. Pain specialist physicians made the pain diagnoses and classifications as part of the clinical treatment programme. The pain conditions were categorised into four etiological groups: neuropathic, nociceptive, visceral, and idiopathic pain. In the case of more than one chronic pain condition, the main presenting pain was chosen as the primary one.

### Data Analysis

Means, standard deviations, distributions, and frequencies for all variables were calculated. Cronbach’s alpha was used to measure internal consistency of the sum scales. Pearson`s correlation coefficient was used to test the associations between the continuous variables. As the analysis of the study utilised the BDI factor model proposed by Morley and colleagues, we conducted a confirmatory factor analysis (CFA) in order to test whether our data fitted the model.

Comparison was made between patients with or without the DSM-IV-based diagnosis of current major depressive disorder (MDD). Student`s t-test was used for continuous variables and the Mann-Whitney U test for non-parametric data. Due to multiple comparisons, the alpha level was corrected according to the Bonferroni correction or according to the recommendations concerning multiple correlated tests [26, 27]. Logistic regression analysis was used to assess the association of the BDI scores and the pain-related variables with the diagnosis of MDD. In Model 1 the association of pain severity with the diagnosis of MDD was assessed after controlling for age and gender. In model 2 the BDI factors were added to the model. Model 3 assessed whether pain disability affects the model. A small number of missing values (2/100 for pain intensity and 3/100 for pain disability) were replaced by the mean of the variable. The analyses were performed using the SPSS/PASW (Predictive Analytics SoftWare) statistical package and AMOS (Analysis of Moment Structures) [28].

## Results

Sixty-two (62%) of the patients were female. Mean age was 47.9 years (SD 7.32, range 30–60). Sixty percent of the patients were married or cohabiting. Twenty-five percent had no professional education, 54% had a vocational education, and 21% had a university level education. Thirty-nine percent were employed and working, 39% on sick leave, 12% in receipt of a disability pension, and 4% unemployed. The median duration of pain was 4 years (range 1–44 years). Forty-nine percent of the patients were classified as having neuropathic pain, 21% nociceptive pain, 5% visceral pain, and 25% idiopathic pain. Descriptive statistics for the pain measures and psychological variables are presented in Table 1. More detailed information concerning patient demographics, pain characteristics, and psychiatric disorders is presented in the previous articles describing the same patient group [29, 30]. The CFA indicated that the fit between the data and the BDI factor model of Morley et al. [17] was acceptable. As an indication of good fit, the chi-squared 74.4, with 64 degrees of freedom, provided a non-significant p-value (0.18). The RMSEA (Root Mean Square Error of Approximation which measures discrepancy per degree of freedom) was 0.041 (95% CI 0.00 to 0.075), when a value of less than 0.05 is taken to indicate a good fit.

**Table 1:** Descriptive statistics for pain and psychological variables.

	Minimum	Maximum	Mean	SD	Cronbach’s alpha
Visual analogue scale of current pain intensity, 0–10mm	0.0	9.9	6.0	2.1	-
BDI sum score, range 0–63 (21 items)	1.0	46.0	17.4	10.3	0.90
BDI, negative view of self score, range 0–18 (6 items)	0.0	18.0	3.9	4.0	0.84
BDI, somatic/ physical function score, range 0–21 (7 items)	1.0	20.0	7.2	3.5	0.73
Pain disability score, range 18–54 (18 items)	22.0	54.0	40.1	7.1	0.87

BDI = Beck Depression inventory

The zero-order correlations are shown in Table 2. The current pain intensity was positively correlated with the BDI somatic/physical function score, as well as with the pain disability score. Pain disability correlated with the BDI somatic/physical function score but not with the BDI negative view of self-score. Twenty patients (20%) fulfilled the current criteria of major depressive disorder. There were no differences of mean age or gender distribution between those with or without MDD.

**Table 2:** Pearson`s zero-order correlation coefficients for pain intensity and psychological variables.

	Current pain intensity, VAS	BDI total	BDI negat.	BDI som./phys.
BDI total	0.15			
BDI negative view of self	0.07	0.84**		
BDI somatic/physical function	0.24*	0.87**	0.55**	
Pain disability	0.23*	0.19	0.09	0.23*

* = p < 0.05,

** = p < 0.001

BDI = Beck Depression Inventory, VAS = Visual Analogue Scale (0–10).

The S1 Table presents the comparisons of the BDI items between patients with and without MDD. Because of the multiple comparisons of the 21 items, the significance of the p-value was adjusted to 0.0034, according to recommendations concerning correlated variables [26, 27]. Patients with MDD had higher scores in several of the BDI items. The greatest differences between the MDD and the non-MDD groups were in the items of the somatic/physical function factor as well as the items not included in the model. Insomnia and weight loss were least associated with the depression diagnosis. Among the items in the negative view of self-factor, BDI 5 ‘guilt’ was the only item to differ significantly between the groups. The patient groups differed significantly also on the levels of the BDI sum scores. The mean BDI total score in patients without MDD was 14.5 (8.1) and in those with MDD 29.0 (9.9). The sum scores derived from the BDI items not in the model differed also between patients with and without MDD. Patients with MDD had higher pain intensity than those without. However, the pain disability score did not differ between the groups (Table 3). In the logistic regression analysis (Table 4), the current pain intensity and the BDI somatic/physical function scales were associated with the current MDD diagnosis, while the negative view of self and the pain disability scores were not. The analysis was also performed adding the negative view of self-scale first (controlling gender, age, and pain intensity) and then the somatic/physical scale. The negative view of self-scale had a significant association with MDD (Wald 9.90, p = 0.002, OR (95%CI) 1.25 (1.10–1.44)), but after adding the somatic/physical scale, the association became non-significant.

**Table 3:** Comparison of the BDI and pain scales by the diagnosis of Major Depressive Disorder (MDD).

	Without MDD (n = 80) mean (SD)	MDD (n = 20) mean (SD)	t	df	p
BDI Negative view score	3.1 (3.4)	7.1 (4.9)	-3.51	24	0.002
BDI Somatic/Physical function score	6.2 (2.6)	11.2 (4.0)	-6.86	98	< 0.001
BDI Items not in the model score	5.3 (3.5)	10.7 (3.8)	-6.12	97	< 0.001
BDI total score	14.5 (8.1)	29.0 (9.9)	-6.82	98	< 0.001
Current pain, VAS 0–10mm	5.7 (2.1)	7.1 (1.6)	-2.82	98	0.006
Pain disability score	39.6 (7.0)	42.6 (7.2)	-1.62	95	0.11

BDI = Beck Depression Inventory

**Table 4:** Logistic regression analysis with current Major Depressive Disorder (MDD) as dependent variable.

	Model 1				Model 2				Model 3			
Variable	B	S.E.	p	OR (95%CI)	B	S.E.	p	OR (95%CI)	B	S.E.	p	OR (95%CI)
Age	0.01	0.04	0.72	1.01 (0.94–1.10)	-0.01	0.05	0.84	1.01 (0.91–1.12	0.03	0.05	0.55	1.03 (0.93–1.14)
Gender	0.75	0.61	0.22	2.11 (0.64–6.97)	1.67	0.89	0.06	5.31 (0.93–30.3)	1.60	0.86	0.063	4.94 (0.92–26.7)
Current pain intensity	0.50	0.18	0.005	1.64 (1.16–2.33)	0.42	0.22	0.06	1.53 (0.98–2.37)	0.47	0.24	0.047	1.60 (1.01–2.53)
BDI Negative view of self					0.07	0.10	0.45	1.07 (0.89–1.29)	0.06	0.09	0.54	1.06 (0.88–1.27)
BDI Somatic/ physical function					0.54	0.15	0.001	1.72 (1.26–2.35)	0.52	0.16	0.001	1.69 (1.23–2.31)
Pain disability									-0.04	0.06	0.55	0.97 (0.86–1.08)
R^2^	0.097				0.34				0.35			

Model 1 = the association of pain intensity with the diagnosis of MDD assessed after controlling for age and gender.

Model 2 = BDI Factor variables added to the model.

Model 3 = Pain disability added to the model.

BDI = Beck Depression Inventory

B = standardized coefficient

## Discussion

Assessment of depression in chronic pain has been challenging because several somatic symptoms overlap between depression and pain. In the present study, we compared two different approaches of assessing depression in chronic pain, the DSM-IV MDD diagnosis and BDI, using the two-factor model recommended by Morley and colleagues [17]. The data fitted the presumed two-factor model in the confirmatory factor analysis, supporting the idea of distinct factors of BDI being related to chronic pain.

Patients with current MDD had higher scores in several of the BDI items than those without MDD. The items that were least associated with the depression diagnosis, such as insomnia, somatic preoccupation, and weight loss, may be considered to reflect the general distress attributable to a somatic disease. Concerning the two-factor model, the somatic/physical factor was more strongly related to MDD, four items of seven differing between the groups. The scores of the items that were not in the factor model showed a consistent pattern: patients with MDD had higher scores in most of these. The results give some support to the suggestion that there is less self-negativity in chronic pain-related depression. The regression analysis emphasises the role of somatic symptoms in pain-related depression. One conclusion is that the somatic symptoms have causal relevance in the diagnosis of MDD in pain patients. However, another explanation for the association can be the several somatic symptom criteria of MDD. The symptom overlap phenomenon thus concerns both the BDI and the diagnostic assessment.

The pain disability measure correlated positively with the somatic/physical depression symptoms as well as with current pain intensity. However, it was not associated with the diagnosis of depression. This is contrary to the common finding of depression worsening the functional disability in pain patients [31]. One explanation can be that the disability scale used in the study measures mainly the physical daily activities omitting several other aspects of pain-related disability such as social or cognitive functioning.

According to the DSM-IV diagnostic guidelines for depression, somatic symptoms should be excluded if they are “clearly and fully attributable to the somatic condition” [32]. Even if DSM-IV is considered as the gold standard for assessing depression, the complex and multifaceted nature of chronic pain makes the decisions difficult or even impossible and they rely therefore also on the subjective interpretations of the examiner. In addition, as DSM-IV allows a wide combination of symptoms within MDD, the clinical picture of depression is heterogeneous. Self-blame or negative view of self-form only one item out of nine in DSM-IV, and the diagnosis of MDD is plausible even in the absence of these symptoms. The recently published DSM-V did not give any further advice how to assess the somatic symptoms of depression [32].

The various criteria of depression may lead into problems of specificity. On the other hand, excluding somatic symptoms in the diagnosis of depression may drastically reduce the rate of diagnosis [33]. Using strict diagnostic criteria in the context of a somatic illness reduces the sensitivity, leading to a missed diagnosis and prolonged symptoms of depression [6]. From the clinical point of view, false negative results in depression assessment may have more serious consequences than overdiagnosis. Comorbid depression has been associated with an increased risk of suicidality in chronic pain [34].

This study has several limitations. The number of patients was low which reduced the statistical strength of the study in general. The number of patients who fulfilled the criteria of current MDD was too low to enable a separate factor analysis of BDI in this group. The discrepancies between the depression findings defined by the factor model and the MDD can partly be attributed to the low number of patients with MDD. We performed the entire SCID interview with all the patients; however, we used only the current major depressive disorder section in the analysis. The study was performed in a tertiary pain clinic, and the patients therefore represent a highly selected group of chronic pain patients. The pain disability measure of the study is widely used in clinical practice in the Pain Clinic of Helsinki University Central Hospital. The measurement is straightforward and covers broadly different areas of functional disability. However, it has not been largely used in previous studies and its validity and reliability need further assessment. Excluding chronic pain patients prescribed strong opioids may have excluded those with the most challenging pain problems.

In conclusion, analysing separately the somatic and cognitive-emotional symptoms can shed more light on the depression concept in chronic pain. The important question for future research is whether the different symptoms of depression associate differently with other elements such as disability, pain intensity or treatment outcome. Longitudinal studies are needed to clarify further these mechanisms. These studies could address the temporal association of depression and pain both when these comorbidities develop and resolve. The assessment of depression in chronic pain remains a challenge even if one utilises the standardised diagnostic systems such as DSM. Considering the symptom overlap problem from the clinical point of view, one option is to at least partly accept the indistinct boundaries between chronic pain, distress, and depression. Combining different methodologies and assessment tools may help to understand the multifaceted nature of depression and its boundaries with chronic pain.

## Supporting Information

S1 Table Comparison of BDI items in patients with and without MDD. (DOC)
