# Supplementary material for: Diagnosing Depression in Chronic Pain Patients: DSM-IV Major Depressive Disorder vs. Beck Depression Inventory (BDI)
Source: PLoS One. 2016 Mar 23;11(3):e0151982. doi: 10.1371/journal.pone.0151982 (PMC4805196; doi:10.1371/journal.pone.0151982)
Supplement: S1 Table — (DOC) [file pone.0151982.s001.doc]

| Table S1. Comparison of BDI items in patients with and without MDD | | | | | |
| --- | --- | --- | --- | --- | --- |
|  | Without MDD  (n=80) | MDD  (n=20) |  |  |  |
|  | Mean Rank | Mean Rank | Mann-Whit. *U* | Z | p^a^ |
| BDI Negative view of self |  |  |  |  |  |
| BDI 3 Sense of failure | 47.87 | 61.03 | 589.50 | -2.09 | .037 |
|
| BDI 5 Guilt | 46.43 | 66.80 | 474.00 | -3.27 | .001* |
|
| BDI 6 Punishment | 47.66 | 61.88 | 572.50 | -2.62 | .009 |
|
| BDI 7 Self-dislike | 47.12 | 64.03 | 529.50 | -2.78 | .006 |
|
| BDI 8 Self-accusation | 47.43 | 62.80 | 554.00 | -2.30 | .022 |
|
| BDI 14 Body image change | 46.52 | 63.75 | 515.00 | -2.65 | .008 |
|
| BDI Somatic/physical function |  |  |  |  |  |
| BDI 12 Social withdrawal | 43.80 | 77.30 | 264.00 | -5.06 | <.001* |
|
| BDI 15 Work difficulty | 47.06 | 64.28 | 524.50 | -2.76 | .006 |
| BDI 16 Insomnia | 48.39 | 58.95 | 631.00 | -1.60 | .11 |
| BDI 17 Fatigability | 45.77 | 69.43 | 421.50 | -3.64 | <.001* |
|
| BDI 18 Loss of appetite | 45.54 | 70.33 | 403.50 | -4.18 | <.001* |
| BDI 20 Somatic preoccupation | 48.01 | 60.48 | 600.50 | -2.09 | .037 |
|
| BDI 21 Loss of libido | 44.86 | 73.08 | 348.50 | -4.07 | <.001* |
| Items not in the model |  |  |  |  |  |
| BDI 1 Sadness | 46.15 | 67.90 | 452.00 | -3.28 | .001* |
| BDI 2 Pessimism | 45.53 | 70.38 | 402.50 | -3.61 | <.001* |
| BDI 4 Dissatisfaction | 45.68 | 69.80 | 414.00 | -3.63 | <.001* |
| BDI 9 Suicidal ideas | 45.33 | 71.20 | 386.00 | -4.11 | <.001* |
|
| BDI 10 Crying | 47.68 | 61.80 | 574.00 | -2.21 | .027 |
|
| BDI 11 Irritability | 44.86 | 73.05 | 349.00 | -4.51 | <.001* |
| BDI 13 Indecisiveness | 44.74 | 73.55 | 339.00 | -4.25 | <.001* |
| BDI 19 Weight loss | 47.94 | 58.15 | 627.00 | -1.77 | .076 |

^a^ p-value significance level adjusted to p < 0.0034 according to the recommendations concerning correlated variables (Li and Ji, 2005; Nyholt, 2004)
